# Attribute development and level selection for a discrete choice experiment to elicit the preferences of health care providers for capitation payment mechanism in Kenya

**DOI:** 10.1186/s13561-019-0247-5

**Published:** 2019-10-30

**Authors:** Melvin Obadha, Edwine Barasa, Jacob Kazungu, Gilbert Abotisem Abiiro, Jane Chuma

**Affiliations:** 1 Health Economics Research Unit, KEMRI | Wellcome Trust Research Programme, P.O. Box 43640 – 00100, Nairobi, Kenya; 2 Nuffield Department of Medicine, University of Oxford, Oxford, UK; 3 Department of Planning, Faculty of Planning and Land Management, University for Development Studies, Wa, Ghana; 4 World Bank Group, Kenya Country Office, P.O. Box 30577-00100, Nairobi, Kenya

**Keywords:** Attribute development, Capitation, Discrete choice experiment, Kenya, Provider payment mechanisms, Sub-Saharan Africa

## Abstract

**Background:**

Stated preference elicitation methods such as discrete choice experiments (DCEs) are now widely used in the health domain. However, the “quality” of health-related DCEs has come under criticism due to the lack of rigour in conducting and reporting some aspects of the design process such as attribute and level development. Superficially selecting attributes and levels and vaguely reporting the process might result in misspecification of attributes which may, in turn, bias the study and misinform policy. To address these concerns, we meticulously conducted and report our systematic attribute development and level selection process for a DCE to elicit the preferences of health care providers for the attributes of a capitation payment mechanism in Kenya.

**Methodology:**

We used a four-stage process proposed by Helter and Boehler to conduct and report the attribute development and level selection process. The process entailed raw data collection, data reduction, removing inappropriate attributes, and wording of attributes. Raw data was collected through a literature review and a qualitative study. Data was reduced to a long list of attributes which were then screened for appropriateness by a panel of experts. The resulting attributes and levels were worded and pretested in a pilot study. Revisions were made and a final list of attributes and levels decided.

**Results:**

The literature review unearthed seven attributes of provider payment mechanisms while the qualitative study uncovered 10 capitation attributes. Then, inappropriate attributes were removed using criteria such as salience, correlation, plausibility, and capability of being traded. The resulting five attributes were worded appropriately and pretested in a pilot study with 31 respondents. The pilot study results were used to make revisions. Finally, four attributes were established for the DCE, namely, payment schedule, timeliness of payments, capitation rate per individual per year, and services to be paid by the capitation rate.

**Conclusion:**

By rigorously conducting and reporting the process of attribute development and level selection of our DCE,we improved transparency and helped researchers judge the quality.

## Introduction

Stated preference elicitation methods such as discrete choice experiments (DCEs) are now being widely used in health preference research in areas such as priority setting, health workforce, and valuation of health outcomes among others [1–4]. A DCE is an econometric technique used to elicit the preferences for the characteristics (attributes) of goods or services [5]. Respondents in a DCE survey are given two or more distinct alternatives to choose from. The alternatives are described by two or more attributes [6]. From the choices made in a DCE survey, researchers can determine the relative importance respondents place on the attributes of the goods or services under consideration, and trade-offs study participants are willing to make on one attribute over another [7].

Theoretically, DCEs draw from Lancaster’s theory of consumer demand and Random Utility Theory (RUT). Lancaster’s theory states that individuals derive utility from the attributes of the good or service rather than the product itself [8]. RUT posits that individuals are rational decision makers and will choose the alternative that they derive the maximum or highest utility from [9].

However, the “quality of DCEs has been questioned” and the way they are designed due to underreporting of the design process [10, 11]. Researchers fail to rigorously conduct and report some aspects of the DCE design process such as attribute development and level selection [11–13]. This may lead to misspecification of attributes and levels which may in turn give erroneous results and hence misinform policy [14]. Therefore, it is important to meticulously conduct and report the process of attribute development and level selection to improve transparency and help researchers judge the quality of the DCE [12, 15].

Researchers need to comprehensively report:

the processes used to collate an initial list of attributes, the analyses conducted during this design stage (including sample details and information on type of analysis conducted), processes undertaken in reducing attributes to a manageable number, and a brief description of the results of these processes [12] (p2).

However, this is complicated by the lack of a standardised process to guide the selection of attributes and levels for health related DCEs [16]. Although guidelines on how to conduct health-related DCEs exist [17–19], they do not provide comprehensive guidance on how to select attributes and levels [12, 16]. Researchers are therefore left to superficially select attributes and levels and vaguely report the process [10, 20]. Nonetheless, few researchers have recently formulated guidelines on how to report the attribute development and level selection process of health-related DCEs [12, 21]. Furthermore, an increasing number of health-related DCEs are now starting to rigorously report the attribute development and level selection process. Examples include DCEs on micro health insurance in Ghana [14], basic health insurance in Iran [22], cataract surgery in Australia [23], and antirheumatic drugs in the Netherlands [24].

We address these research gaps and contribute to the limited literature on attribute development and level selection by rigorously conducting and reporting the process followed in deriving attributes and levels for a DCE to elicit the preferences of health care providers for the attributes of capitation payment mechanism in Kenya. Capitation is a provider payment mechanism (PPM) used by purchasing organisations (e.g. health insurance companies, governments) to pay health care providers to deliver services to people [25]. It is a fixed payment made to a health care provider in advance to extend services to enrolled individuals for a period of time [25].

PPMs are important as they have the potential to modify health care provider behaviour and influence providers to deliver needed services, improve quality, and efficiency [26]. For example, capitation creates incentives for providers to improve efficiency, contain costs, increase number of enrolees, select healthy individuals, and underprovide health services [25, 27]. In Kenya, capitation is used by the country’s National Hospital Insurance Fund (NHIF) to pay for outpatient services for its enrolees at contracted public, private, and faith-based facilities [28, 29].

Since PPMs can create positive and negative incentives, it is important to consider health care providers’ preferences for their design attributes. A DCE is the right technique as it will enable the eliciting of health care providers’ preferences for the attributes of capitation, quantification of the relative importance providers place on the characteristics, and trade-offs respondents are willing to make [7]. These attributes can be targets for potential interventions meant to configure capitation payment mechanisms to create positive incentives for health care providers and help to steer the health system towards universal health coverage (UHC) [30]. However, there is a dearth of literature on DCEs that have focussed on health care providers preferences for capitation payment methods in low-middle income countries (LMICs) with the exception of Robyn et al. [31].

The aim of this paper was to describe the techniques used to derive the initial set of attributes and levels, methods employed in reducing the number of attributes and selecting levels, piloting, and concluding discussions to decide on the final list of attributes and levels.

## Methodology

### Conceptual framework

**Fig. 1:**
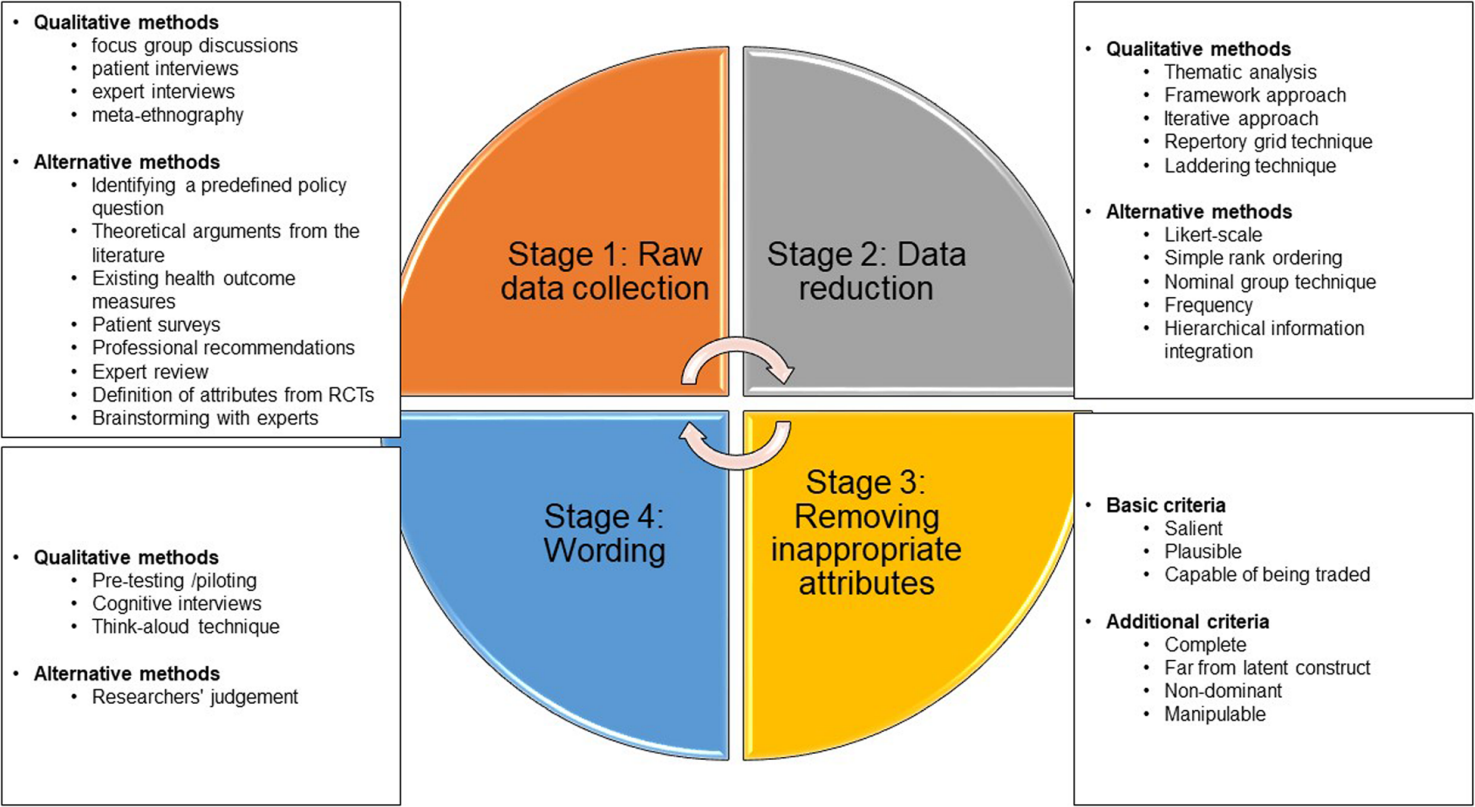
Conceptual framework for attribute development and level selection. Adapted from Helter and Boehler [21]

We applied a framework proposed by Helter and Boehler [21] (Fig. 1). The researchers provide a systematic approach to attribute development for health-related DCEs and recommend following a four-stage process consisting of raw data collection, data reduction, removing inappropriate attributes, and wording of attributes.

First, raw data about attributes and levels are collected using qualitative studies and alternative methods such as literature reviews. Then, the collected data are reduced through analysing. This results in a long list of attributes and levels. These are then screened for appropriateness considering multiple criteria such as salience, plausibility and capability of being traded, to reduce them to a limited number of attributes and levels. Finally, the attributes and levels are worded using methods such as piloting, cognitive interviews or researchers’ judgement.

#### Stage 1: raw data collection

To derive an initial list of attributes and levels, a literature review and a qualitative study were conducted. These were guided by a framework developed by the Resilient and Responsive Health Systems (RESYST) consortium on the characteristics of multiple funding flows to health facilities (Table 1) [32]. Using both a literature review and a qualitative study is recommended as the former generates conceptual attributes while the latter unearths context-specific characteristics [11, 14].

**Table 1:** Framework for the characteristics of capitation payment mechanism

Attribute	Definition	Levels
1. Adequacy or sufficiency of the payment rate	The extent to which the payment rate covers the costs of services purchased	Adequate Inadequate
2. Predictability of payment amounts	Whether providers know what amount to expect	Predictable Unpredictable
3. Predictability of payment patterns	Whether providers know when they will get paid	Predictable Unpredictable
4. Complexity of the accountability mechanisms associated with the PPM	The complexity of the accountability and reporting mechanisms associated with the PPM	Simple accountability requirements Complex/burdensome accountability requirements
5. Service coverage of the PPM	The range of services the PPM is paying for	Outpatient services, inpatients services, dental, optical, surgical, nursing care
6. The performance requirements of the PPM	Whether the PPM is tied to performance	Payments tied to performance Payments not tied to performance
7. Flexibility or autonomy of the PPM	Autonomy health care providers have to spend or use the PPM funds on anything	Autonomous (Flexible) Restricted (Rigid)

Adapted from RESYST consortium’s framework on the characteristics of multiple funding flows [32]

##### Literature review

The literature review sought to synthesise evidence on the characteristics of PPMs that influenced health care provider behaviour. The search was conducted using three databases namely PubMed, Web of Science, and Google scholar. Search terms such as “provider payment mechanisms”, “capitation”, fee-for-service”, “remuneration methods” among others were used. Full text peer reviewed journal articles that had been published in English by February 2018 and described empirical research on PPMs were eligible. Papers that described incentives that modified health care provider behaviours were excluded. Two researchers independently screened the articles.

##### Qualitative study

A cross sectional qualitative study was conducted in two Kenyan counties. The study sought to explore the experiences of health care providers with PPMs in the Kenyan context and examined the characteristics of these payment methods that providers considered important. The framework for the characteristics of capitation (Table 1) was used. First, two counties were purposively sampled. Then, six NHIF accredited providers (two private, two public, two faith-based) were purposively selected. Next, institutional heads of the health facilities were approached using emails, phone calls, and face to face visits and consent sought to participate in the study. After that, five senior managers and health management team members (HMT) whose roles involved financial decision making were selected in each facility. Of the 30 respondents approached, one senior manager at a private health facility declined to participate citing a busy schedule.

Overall, 29 semi-structured interviews were conducted with respondents at their workplace after obtaining written informed consent. The respondents had diverse management roles from medical directors to financial managers (Table 2). Data were collected between September and December 2017. The interview guide (Additional file 1) was developed by three researchers using the framework for the characteristics of capitation (Table 1) and explored areas such as awareness and understanding of PPMs, experiences with capitation and FFS, attributes of PPMs they considered important, and attribute levels of capitation and FFS. Furthermore, respondents were prompted to spontaneously mention the characteristics of an ideal PPM and rank them in the process. The guide was tested in one county at different health facilities. The interviews were audio recorded, lasted between 30 and 50 min, and conducted in English. The interviewers wrote field notes during and after the interviews.

**Table 2:** Characteristics of qualitative study respondents

Interview respondent	Number
Medical Directors and Superintendents	6
Pharmaceutical personnel	6
Administrative officers and directors	6
Nurses-in-charge	6
Clinical officers-in-charge	1
Financial Managers and Accountants	4
Grand total	29

#### Stage 2: data reduction

##### Literature review

Overall, 27,156 papers were found. We excluded 27,012 papers because they did not meet the inclusion criteria by reading the titles. Then, abstracts of 144 papers were read resulting in 93 articles being excluded for not meeting the criteria. Thereafter, a further 20 papers were excluded due to unavailability of full text articles. The resulting 31 papers were read in full and 15 duplicates were dropped. The review finally included 16 papers. The literature review has been published [33].

##### Qualitative study

A framework approach was used in qualitative data analysis. The interviews were first transcribed verbatim in full. Then, two researchers familiarised themselves by reading and rereading the transcripts. The coding framework was developed by three researchers from the framework on the characteristics of capitation, study objectives, and emerging themes. This process culminated in a coding tree. The coding tree touched on attributes and attribute-levels of capitation. NVIVO version 10 was used to manage the data [34]. One researcher applied the codes, sorted, and conducted the charting. Finally, three researchers interpreted the findings. The qualitative study has also been published [30].

#### Stage 3: removing inappropriate attributes

##### Panel of experts

To reduce the list of attributes and levels, we engaged a panel of eight experts that comprised of doctors, nurses, pharmacists, and researchers. It is a recommended method when one needs to reduce the number of attributes and levels [17]. Too many attributes in a DCE increase complexity of the tasks for the respondents which, in turn, result in increased error variance, attribute non-attendance (a phenomenon where not all attributes are considered in reaching a decision), and inconsistent responses across choice tasks [5, 35, 36].

The experts had experience working in similar settings (health facilities) as the potential DCE respondents. Therefore, they could provide valuable feedback on the attributes and levels that would mirror those of DCE respondents. The experts and researchers together screened all the capitation attributes and levels generated from the data reduction stage. They used multiple criteria such as relevance to study objectives and decision context, correlation between attributes (inter-attribute correlation), salience, plausibility, and capability of being traded [17, 21].

##### Researchers’ judgement

Three researchers (authors) held two meetings to review the decisions of the experts. They also agreed on an interim list of capitation attributes and levels to be included in a pilot study.

#### Stage 4: wording

##### Pilot study

A pilot study was conducted to pre-test the interim list of attributes and levels that had been agreed upon by the authors. Moreover, we also aimed to generate parameter estimates that would be used to construct an appropriate experimental design for the main DCE survey. For the pilot study, a D-efficient experimental design was generated using the Ngene software version 1.2.0 [37]. It entailed an unlabelled experiment with two alternatives and an opt-out (no-choice alternative). We used educated best guesses to generate the priors [38]. Eight full profile choice tasks were derived and transferred to a paper questionnaire (Table 3 and Additional file 2). Since the DCE targeted senior managers who were often busy, eight choice tasks would not place significant cognitive burden on the respondents.

**Table 3:**
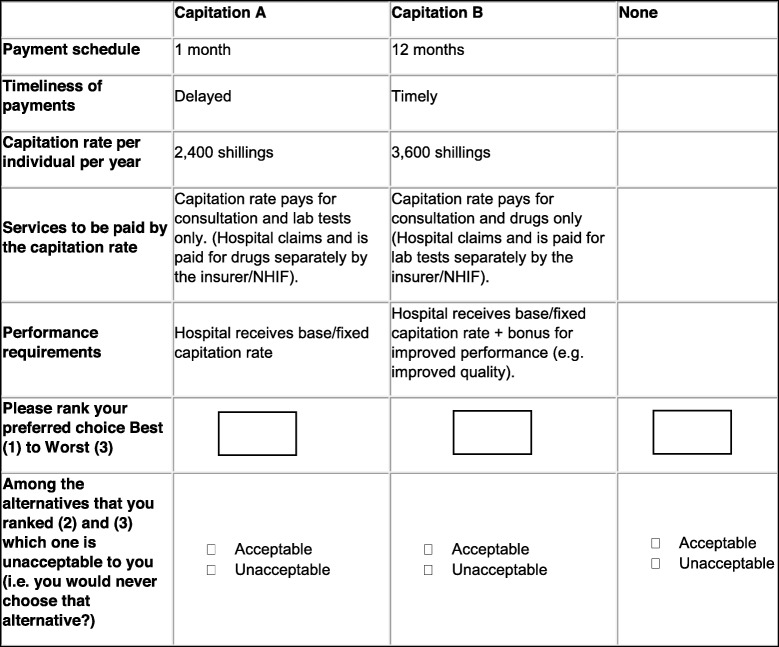
Sample DCE pilot choice task

The pilot study questionnaire (Additional file 2) was administered to 31 senior managers and members (Table 4) from 9 randomly selected public, private, and faith-based health facilities in one Kenyan county (83.78% response rate) [39]. Respondents were prompted to rank their preferences from best (1) to worst (3) considering two hypothetical capitation payments (Capitation A and B) and an opt-out (no-choice alternative labelled ‘none’) (Table 3). Furthermore, respondents were also required to specify which options they found unacceptable to them i.e. they would never choose (a no concession outcome). The main aim of this was to approximate decisions made by groups using a technique called minimum information group inference (MIGI) [40, 41]. Moreover, we asked study participants for general feedback on the choice tasks, understandability of the scenarios, questionnaire design, appropriateness, wording, and clarity of the attributes and levels. A think aloud approach was also employed where respondents were asked to verbalise their thought process when answering the choice tasks [12, 21]. Data was collected between May and June 2018.

**Table 4:** Characteristics of pilot study respondents

Characteristic	Proportion	N
Sex		
Male	58.06%	18
Female	41.94%	13
		31
Job titles		
Medical directors and superintendents	19.35%	6
Pharmaceutical personnel-in-charge	9.68%	3
Administrative officers and directors	19.35%	6
Nurses-in-charge	16.13%	5
Clinical officers-in-charge	9.68%	3
Financial managers and accountants	16.13%	5
Medical laboratory personnel-in charge	3.23%	1
Medical social workers	3.23%	1
Chief executive officers	3.23%	1
		31
Type of health facility the respondent works in		
Public	35.48%	11
Private-for-profit	29.03%	9
Faith-based & NGOs	35.48%	11
		31
level of care the respondent worked in		
Primary care	12.90%	4
Secondary care	87.10%	27
		31
Total work experience		
Mean in years (standard deviation)	13.32 (12.57)	31
Median in years (inter-quartile range)	9 (4.5, 15.5)	31
Age		
Mean in years (standard deviation)	39.81 (12.71)	31
Median in years (inter-quartile range)	34 (30.5, 42.5)	31

A multinomial logit model (MNL) was used to estimate individual preferences on R version 3.5.0 using the University of Leeds Choice Modelling Centre’s (CMC) choice modelling code for R (cmcRcode) version 2.0.4 [42, 43]. We estimated the main effects. Willingness to accept (WTA) measures were also estimated from the MNL model coefficients using the delta method. Additionally, the relative importance scores were derived from the MNL model coefficients [44]. This was done through multiplying the absolute value of the coefficient of each attribute with the difference between the highest and lowest level of the attribute to get the maximum effect. Then, the ratio between the maximum effect of each attribute and the total was computed to derive the relative importance scores [44]. Finally, to test the robustness of our results and relax the Independence of Irrelevant Alternatives (IIA) property, we also estimated a mixed multinomial logit model (Additional file 4) [45].

##### Researchers’ final discussions

Six researchers reviewed the results of the pilot study, respondents’ comments, and made amendments to the DCE questionnaire. They then agreed on the final list of attributes and levels for the main DCE survey.

## Results

### Results from stages 1 and 2: raw data collection and data reduction

The literature review found that seven PPM characteristics influenced health care provider behaviour (Table 5).

**Table 5:** Attributes of PPMs

Attributes	
1. Accountability mechanism [46–50]	
2. Bundling of services [51–53]	
3. Payment rate [31, 47, 49, 51, 53–57]	
4. Payment schedule [31, 46, 49]	
5. Performance indicators [48, 49, 57–59]	
6. Sufficiency of payment rate [31, 51, 52, 60]	
7. Timeliness of payment [47, 52, 56]	

Source: Kazungu et al. [33]

Semi-structured interviews with senior managers and HMT members uncovered 10 attributes of capitation that health care providers considered important (Table 6).

**Table 6:** Capitation attributes

Attribute	Definition	Levels	Quotes
1. Adequacy of the payment rate to cover the cost of services.	Adequacy of the capitation rate to cover the costs of the services provided to the individual/enrolee.	Adequate to cover the costs. Inadequate (Patients must co-pay) Inadequate (Patients don’t co-pay)	*“[The rate should be adequate] to cover 100%.... The patient pays nothing at all.” - HMT member 3 | public provider (County B)* *“Well if the NHIF is not able to top-up for the patient, then the patients should top-up for themselves.” – Senior manager 4 | faith-based provider (County B)* *“They should not pay because this patient might have paid for that insurance for ten years and he is becoming sick now. Surely, they should not pay anything else.” - HMT member 1 | public provider (County B)*
2. Services covered	Services to be paid by the capitation rate.	All services including complex diagnostics e.g. imaging, optical and dental services. Consultation + laboratory tests + drugs. Consultation + drugs Consultation + laboratory tests. Laboratory tests + drugs Consultation + other diagnostics Consultation only	*“I think it should include consultation, lab work, medication, [and] something like X-ray. They should be in that capitation.” – Senior manager 2 | private provider (County B)* *“At least it should cover all the out-patient services, lab, pharmacy, [and] consultation.” - Senior manager 5 | faith-based provider (County B)*
3. Autonomy to use capitation funds.	Freedom the health care provider has in using capitation funds.	Flexible Rigid	*“I think there should be restrictions. Because, you know, money is money.” - Senior manager 5 | private provider (County B)* *“To us, I think they [purchasers] could come up with a method such that I’m allowed to keep my [money]... If I collect my own user fees, let me be allowed to bank it in my own... hospital account other than bank it to the [county] revenue account.” – HMT member 2 | public provider (County B)*
4. Predictability of payments in terms of timing	How predictable the timing of payments is	Timely (Providers know when they will be paid). Delays (Providers don’t know when they will be paid).	*“We would prefer getting these moneys quarterly. And if the decision has been made that this money is to reach the facility quarterly, then for heaven’s sake, let it be quarterly” – HMT member 4 | public provider (County A)*
5. Payment schedule	Timing of payment disbursements	2 weeks 4 Weeks (Monthly) 3 months (Quarterly) 6 months (Bi-annually) 12 months (Annually)	*“After every two weeks it would better.” – Senior manager 2 | private provider (County A)* *“On quarterly basis, the way they [NHIF] have been doing [it]… because we make our budgets after every three months.” – HMT member 1 | public provider (County A)*
6. Predictability of payments in terms of amount	How predictable the payment amounts are	Predictable – (Providers know the amount to expect) Unpredictable (Providers don’t know the amount to expect).	*“I wish to know what the value is. If I sold you a car for KES 100,000 [US $ 1000], I should be getting my 100,000. I’m not expecting 150 for it. Or I am not expecting 80,000 for it. I get my money. That is what I am expecting, isn’t?” - Senior manager 4 | private provider (County B)*
7. Capitation amount	Payment rate per individual/enrolee per year	1200 per individual per year 1500 per individual per year 2000 per individual per year 2400 per individual per year 3000 per individual per year 3600–4800 per individual per year 4000 per individual per year 5000 per individual per year 6000 per individual per year 10,000 per individual per year 80,000 per individual per year	*“Capitation and what they are paying is a hundred shillings [US $1 per individual per month]. So, if you could make it three - four hundred shillings [US $ 3–4 per individual per month], that would be fine.” – Senior manger 3 |private provider (County A).* *“Five thousand [US $ 50] per person per year” – Senior manager 1 | faith-based provider (County B)* *“For an individual per year under capitation, if I may [mention the] rate, it is 6000 [US $60] in a year.” - Senior manager 1| faith-based provider (County A)*
8. List of clients registered to a health facility	List of people/enrolees registered to a health facility under capitation payments	List available List not available	*“So, I think the best thing is that they [NHIF] are supposed to give us… the list of the patients that we are supposed to treat and the benefits” – Senior manager 4 | private provider (County B)* *“I would prefer us to know the number of clients that have been allocated this facility for outpatient.” - HMT member 1 | public provider (County A)*
9. Complexity of accountability mechanisms	How complex reporting and accountability mechanisms associated with capitation are e.g. notifying the NHIF using online systems every time a patient seeks care at the facility.	Simple accountability requirements Complex/burdensome accountability requirements	*“Simple… so that to enable any health care worker maybe to… sign on that [NHIF] form.” – HMT member 2 | public provider (County A)* *“Yeah, they [NHIF] should be strict … If I am using an NHIF form, I should have proof that this patient is the same one who is being treated. I should confirm the ID number and the age of the patient.” - Senior manager 5 | private provider (County B)*
10. Performance requirements	Whether payment mechanism is tied to performance or not	Payments linked to performance Payments not linked to performance	*“It should be performance-based. If you are good, they pay us good. Isn’t it? If you’re not doing well, they can say no.” – Senior manager 3 | private provider (County B)* *“Performance-based payment is also an incentive for a facility because it will give you more inspiration to work… You know even in the Bible, it’s very clear. When you are given more, even more is expected of you.” - Senior manager 2 | faith-based Facility (County A)* *“Capitation cannot work with performance because not everyone will come to the hospital.”– HMT member 4 | public provider (County B)*

Moreover, senior managers and HMT members spontaneously mentioned the attributes of an ideal PPM while ranking them in the process during the qualitative study. The most important trait of a PPM was timeliness of the payment, followed by services covered by the PPM, adequacy of the payment rate to cover the cost of services, complexity of accountability mechanisms, autonomy that health care providers have over the use of PPM funds, and lastly list of clients registered to a health facility under capitation.

### Results from stage 3: removing inappropriate attributes

#### Panel of experts

The panel discussed all ten capitation attributes from the qualitative study. The attributes from the literature review were conceptual and similar to those unearthed by the qualitative study. The qualitative study had the advantage of being context specific. Three attributes were dropped due to inter-attribute correlation and irrelevance to the decision context (Table 7). The rest were either maintained as they were or reworded. Additionally, the number of levels were capped at four per attribute. Overall, this stage resulted in seven capitation attributes.

**Table 7:** Expert panel’s comments and decisions on capitation attributes and levels

Initial attribute name	Initial levels	Comments by experts	New attribute name	New levels
1. Adequacy of the payment rate to cover the cost of services	Adequate to cover the costs. Inadequate (Patients must co-pay) Inadequate (Patients don’t co-pay)	The attribute was highly correlated with the ‘capitation amount’ attribute. Therefore, the attribute was dropped	Attribute dropped due to inter-attribute correlation	
2. Capitation amount	1200 per individual per year 1500 per individual per year 2000 per individual per year 2400 per individual per year 3000 per individual per year 3600–4800 per individual per year 4000 per individual per year 5000 per individual per year 6000 per individual per year 10,000 per individual per year 80,000 per individual per year	The attribute name was retained as it was salient and would enable calculation of marginal willingness to accept estimates The levels were reduced to four. The currency was in Kenya shillings. The base level was set to “1200” which reflected the current capitation rate per individual per year. Then, to get the other levels 1200 was added to the levels i.e. 1200 + 1200 = 2400. 2400 + 1200 = 3600. 3600 + 1200 = 4800. The levels were plausible and capable of being traded	Capitation amount	1200 per individual per year 2400 per individual per year 3600 per individual per year 4800 per individual per year
3. Services covered	All services including complex diagnostics e.g. imaging, optical and dental services. Consultation + laboratory tests + drugs. Consultation + drugs Consultation + laboratory tests. Laboratory tests + drugs Consultation + other diagnostics Consultation only	The attribute name was retained as it was salient The levels were reduced to three packages namely comprehensive, enhanced and basic. The comprehensive package had all services including complex diagnostic services, optical, and dental services	Services covered	Comprehensive (All services including complex diagnostics e.g. imaging, optical and dental services) Enhanced (consultation + laboratory services + drugs) Basic (Consultation + drugs)
4. Autonomy to use capitation funds.	Flexible Rigid	The attribute was viewed as salient to public health care providers. The panel decided that the terms “flexible” and “rigid” needed to be simplified to be understandable to health care providers. From the qualitative study, this attribute was specific to public providers as they had to first deposit the PPM funds into a pooled account run by the county governments. Then, they would wait for the county governments to reimburse the funds back to them after some time. This was a legal requirement in some counties. Therefore, levels were simplified from “flexible” to “do not have to pay the county first” and “rigid” to “pay the county first as usual.”	Autonomy to use capitation funds.	Do not have to pay the county first. Pay the county first as usual.
5. Payment schedule	2 weeks 4 Weeks (Monthly) 3 months (Quarterly) 6 months (Bi-annually) 12 months (Annually)	The attribute name was viewed as salient and self-explanatory. It was therefore maintained. The level “annual payments (12 months)” was viewed as far apart. Therefore, the level was dropped.	Payment schedule	2 weeks 4 Weeks (Monthly) 3 months (Quarterly) 6 months (Bi-annually)
6. Predictability of payments in terms of timing	Timely (Providers know when they will be paid). Delays (Providers don’t know when they will be paid).	Predictable was viewed as a complex word. Therefore, the attribute name was changed to “payment patterns”. The levels were simplified to include the words “timely” and “delayed” This attribute was viewed to be similar to “payment schedule”. However, it differed from the first one as payment schedule might be set, but not followed.	Payment patterns	Timely Delayed
7. List of clients registered to a health facility	List available List not available	Health care providers in Kenya did not have access to the list of enrolees registered to their health facilities. This was viewed by the panel to be a transparency issue rather than a capitation attribute. Therefore, the attribute was dropped as it was not relevant to the decision context	Attribute dropped as it was not relevant to decision context.	
8. Predictability of payments in terms of amount	Predictable – (Providers know the amount to expect) Unpredictable (Providers don’t know the amount to expect).	Health care providers could not predict the total capitation amount they expected to receive from the purchaser (NHIF) as they did not know the number of clients registered to their health facilities. Therefore, this attribute was deemed to be correlated to the “list of clients registered to a health facility” attribute. Furthermore, it was viewed as a transparency from the purchaser issue rather than a characteristic of capitation. Therefore, the attribute was dropped.	Attribute dropped due to inter-attribute correlation and irrelevance to decision context.	
9. Complexity of accountability mechanisms	Simple accountability requirements Complex/burdensome accountability requirements	The attribute name was maintained as it was salient. Unlike, FFS payments, the main accountability issue with capitation was notifying the NHIF every time an enrolee sought care at a health facility. The levels were simplified by removing the word accountability	Complexity of accountability mechanisms	Simple requirements Complex requirements
10. Performance requirements	Payments linked to performance Payments not linked to performance	The attribute name was maintained as it was salient. It was decided that the attribute and levels should delink individual performance from health facility performance. Since the attribute focussed on health facility performance rather than individual performance, the word “facility” was added to the attribute-levels	Performance requirements	Payments linked to facility performance Payments not linked to facility performance

1 US$ = Kenya shillings (KES) 100

#### Researchers’ judgement

Three researchers held two meetings to deliberate an interim list of attributes and levels that had been agreed by the panel of experts. These were to be included in the pilot study. An agreement was also reached to restrict the maximum number of attributes to five and levels to four per attribute. Five attributes were deemed manageable for the respondents as too many would increase task complexity resulting in increased error variance and attribute non-attendance. Two attributes ‘autonomy to use capitation funds’ and ‘complexity of accountability mechanisms’ were dropped due to irrelevance to the decision context (Table 8). The remaining five attributes and their corresponding levels were simplified, expounded, and reworded.

**Table 8:** Researchers’ comments and decisions on capitation attributes and levels

Initial attribute name	Initial levels	Comments by researchers	New attribute name	New levels
1. Capitation amount	1200 per individual per year 2400 per individual per year 3600 per individual per year 4800 per individual per year	The attribute was renamed ‘capitation rate per individual per year’ to emphasise the fact that the rate stated was for an individual enrolee per year. The levels were simplified by rewording and adding the local currency (shillings).	Capitation rate per individual per year.	1200 shillings 2400 shillings 3600 shillings 4800 shillings
2. Services covered	Comprehensive (All services including complex diagnostics e.g. imaging, optical and dental services) Enhanced (consultation + laboratory services + drugs) Basic (Consultation + drugs)	The attribute was reworded to ‘services to be paid by the capitation rate’ to emphasise the range of services the capitation rate was paying for. The levels expounded to emphasise the fact that providers would not turn away enrolees seeking care if the services were not being paid by the capitation rate. They could claim separately for services not included if they provided them to patients. For example, the insurer might pay for consultation, drugs, and lab tests by capitation, OR pay for consultation and drugs only by capitation, and pay for lab tests separately using another method such as FFS.	Services to be paid by the capitation rate	Capitation rate pays for consultation only. (Hospital claims and is paid for lab tests and drugs separately by the insurer/NHIF). Capitation rate pays for consultation and drugs only (Hospital claims and is paid for lab tests separately by the insurer/NHIF). Capitation rate pays for consultation and lab tests only. (Hospital claims and is paid for drugs separately by the insurer/NHIF). Capitation rate pays for consultation, lab tests, and drugs
3. Autonomy to use capitation funds.	Do not have to pay the county first. Pay the county first as usual.	The attribute was dropped as the issue was not viewed as a PPM characteristic, rather a systemic problem which differed by counties (sub-national regions).	Attribute dropped as it was irrelevant to the decision context	
4. Payment schedule	2 weeks 4 Weeks (Monthly) 3 months (Quarterly) 6 months (Bi-annually)	The attribute name was maintained. As for the levels, two weeks was viewed as short and not plausible for capitation payments. Therefore, it was dropped. Annual payments “12 months” even though had initially been viewed by panel of experts as far apart, was reinstated by the researchers as it thought to be plausible.	Payment schedule	1 month 3 months 6 months 12 months
5. Payment patterns	Delayed Timely	The attribute name was changed to timeliness of payments as it would be easily understandable to the respondents. The levels were maintained	Timeliness of payments	Delayed Timely
6. Complexity of accountability mechanisms	Simple requirements Complex requirements	The attribute name was simplified by deleting the word ‘complexity’. Furthermore, the researchers felt that ‘complexity’ framed the attribute negatively. A note was added to expound on what the levels meant. The attribute was dropped as capitation did not have a reporting mechanism	Attribute dropped as it was irrelevant to the decision context	
7. Performance requirements	Payments not linked to facility performance Payments linked to facility performance	The attribute name was maintained as it was salient The levels were reworded to expound on what performance entailed.	Performance requirements	Hospital receives base/fixed capitation rate Hospital receives base/fixed capitation rate + bonus for improved performance (e.g. improved quality)

### Results from stage 4: wording

#### Pilot study

The previous step resulted in five attributes, namely, payment schedule, timeliness of payments, capitation rate per individual per year, services to be paid by the capitation rate, and performance requirements (Table 9). The levels were then ranked according to expected preferences to enable guess estimating the signs of the attributes. For example, a longer payment schedule would be less desirable. Therefore, the payment schedule attribute was given a negative sign. Furthermore, from the qualitative study, health care providers stated that capitation would not work with performance requirements. For that reason, the performance requirements attribute was given a negative sign.

**Table 9:** Pilot study capitation attributes and levels

Attributes	Attribute type	Levels	Coding	Signs
Payment schedule	Continuous	1 month	1	Negative
		3 months	3	
		6 months	6	
		12 months	12	
Timeliness of payments	Discrete	Delayed	0	Positive
		Timely	1	
Capitation rate per individual per year	Continuous	1200 shillings	1200	Positive
		2400 shillings	2400	
		3600 shillings	3600	
		4800 shillings	4800	
Services to be paid by the capitation rate	Discrete	Capitation rate pays for consultation only. (Hospital claims and is paid for lab tests and drugs separately by the insurer/NHIF)	0	Negative
		Capitation rate pays for consultation and drugs only (Hospital claims and is paid for lab tests separately by the insurer/NHIF)	1	
		Capitation rate pays for consultation and lab tests only. (Hospital claims and is paid for drugs separately by the insurer/NHIF)	2	
		Capitation rate pays for consultation, lab tests, and drugs	3	
Performance requirements	Discrete	Hospital receives base/fixed capitation rate	0	Negative
		Hospital receives base/fixed capitation rate + bonus for improved performance (e.g. improved quality)	1	

We estimated the choice probability for selecting a capitation alternative and willingness to accept (WTA) measures (Table 10). In the preference space, three attributes had statistically significant coefficients namely payment schedule, timeliness of payments, and capitation rate per individual per year. The signs of the estimates were also expected. This meant that capitation alternatives with frequent disbursement schedules, timely payments, and higher rates per individual per year were preferred by the respondents.

**Table 10:** Main effects MNL model estimates

		Preference estimates	Willingness to accept (WTA)
Attributes	Levels	Coefficient (robust se)	value (robust se)
Payment schedule	1 month	− 0.0895** (0.028)	294.3263*** (83.7794)
	3 months		
	6 months		
	12 months		
Timeliness of payments	Delayed	0.4808** (0.1497)	− 1580.4597** (528.7835)
	Timely		
Capitation rate per individual per year	1200 shillings	0.0003** (0.0001)	
	2400 shillings		
	3600 shillings		
	4800 shillings		
Services to be paid by the capitation rate	Capitation rate pays for consultation only.	−0.0360 (0.0833)	118.2276 (270.9732)
	Capitation rate pays for consultation and drugs only		
	Capitation rate pays for consultation and lab tests only		
	Capitation rate pays for consultation, lab tests, and drugs		
Performance requirements	Hospital receives base/fixed capitation rate	0.0540 (0.1085)	− 177.6008 (351.5738)
	Hospital receives base/fixed capitation rate + bonus for improved performance (e.g. improved quality)		
Opt-out		−0.2319 (0.4188)	762.1963 (1423.0172)
Model fit statistics			
Log-likelihood at convergence	− 250.6159		
Log-likelihood (final)	− 222.5633		
Adjusted rho-squared at convergence	0.09		
Akaike Information Criterion	457.13		
Bayesian Information Criterion	478.21		
observations	248		
number of decision makers (n)	31		

s.e. - Robust standard errors in parenthesis. Asterisks denote statistical significance at *** 0.1%, ** 1%, and *5% level

The ‘services to be paid by the capitation rate’ attribute and the opt-out had the expected negative signs but the coefficients were not statistically significant. This might have been due to a small sample size of 31 respondents. Interestingly, the ‘performance requirements’ attribute had an unexpected positive sign. A negative sign was expected according to the qualitative study results which had indicated that senior managers and HMT members would not want performance requirements attached to capitation payment schemes. However, the coefficient was not statistically significant. Nonetheless, when the opt-out was excluded from the analysis (Additional file 3), the coefficient of the ‘performance requirements’ attribute had the expected negative sign. This was also not statistically significant probably due to the small sample size.

The relative importance estimates were derived from the MNL coefficients (Table 11). The most important capitation attribute was payment rate per individual per year followed by payment schedule. The least important was the performance requirements attribute.

**Table 11:** Relative importance estimates

Capitation attribute	Effect	Maximum effect	Relative importance
Payment schedule	0.0895	0.9845	0.3636
Timeliness of payment	0.4808	0.4808	0.1776
Payment rate per individual per year	0.0003	1.0800	0.3989
Services to be paid by the capitation rate	0.0360	0.1080	0.0399
Performance requirements	0.0540	0.0540	0.0199

During the think aloud exercise, respondents raised several issues with the attributes, levels, choice tasks, and questionnaire in general. For example, when respondents were exploring the timeliness of payment attribute (which had 2 levels; timely and delayed), most of them asked for a definition of the length of delay. Study respondents stated that they would accept shorter delays of up to one month for a higher payment rate per individual.

Second, respondents complained that the levels of the ‘services to be paid by the capitation rate’ attribute contained long sentences. For example, a level read as follows; capitation rate pays for consultation and drugs only (Hospital claims and is paid for lab tests separately by the insurer/NHIF). They wanted the levels of the attribute to be simplified by shortening the sentences.

Third, study participants could easily rank the alternatives including the opt-out (no-choice alternative). However, they struggled to understand the second part of the choice question which prompted them to choose the alternative they found unacceptable among those they had ranked second and third (acceptable/unacceptable question). Respondents felt that since they had ranked the alternatives from best (1) to worst (3) in the first part of the choice question, then they would naturally choose the worst ranked alternative as unacceptable in the second part of the task. Furthermore, respondents thought that they were not expected to change which alternative they deemed worst unless there was some form of interaction with other participants’ choices before answering the acceptable/unacceptable question. Overall, the DCE questionnaire took approximately 20 min to complete and the respondents stated that they had sufficient information to make a choice.

##### Final list of attributes and levels

The team of six researchers (authors) made final alterations to the attributes, levels, and choice task design taking into consideration the pilot study results and respondents’ comments. The levels of the ‘payment schedule’ attribute were edited by including a succinct definition of the time periods (Table 12). For example, the word ‘every month’ was added to the ‘1-month’ level to define what it meant.

**Table 12:** Final capitation attributes and levels

Attributes	Levels	Attribute type
Payment schedule	1 month (Every month)	continuous
	3 months (Every quarter)	
	6 months (Twice a year)	
	12 months (Once a year)	
Timeliness of payments	Delayed by more than 3 months	discrete
	Delayed by less than 3 months	
	Timely	
Capitation rate per individual per year	800 shillings	continuous
	1600 shillings	
	2400 shillings	
	3200 shillings	
Services to be paid by the capitation rate	Consultation ONLY	discrete
	Consultation AND Laboratory tests	
	Consultation AND Drugs	
	Consultation AND Laboratory tests AND Drugs AND Imaging (e.g. X-rays)	

Secondly, a level of the ‘timeliness of payments’ attribute was split into two. The ‘delayed’ level was split into two namely ‘delayed by more than 3 months’ and ‘delayed by less than 3 months’. This was in response to the comments raised by the respondents during the pilot study to define the length of the delay.

Thirdly, the ‘capitation rate per individual per year’ attribute had its levels modified. There were some policy considerations to reduce the capitation rate paid to health care providers for the NHIF general scheme. Therefore, the researchers revised the levels to include one that was lower than the current rate of 1200 Kenya shillings (US $ 12). They settled for 800 Kenya shillings (US $ 8). Then, a linear additive value of 800 was added from the base level to get the other three levels. The attribute was maintained as a continuous variable as it was the monetary characteristic that would enable the calculation of willingness to accept estimates.

Moreover, the levels of the ‘services to be paid by the capitation rate’ attribute were simplified by reducing the number of words. For example, the base level was reworded to ‘Consultation ONLY’ from ‘Capitation rate pays for consultation only (Hospital claims and is paid for lab tests and drugs separately by the insurer/NHIF)’.

Furthermore, the pilot study results showed a counter-intuitive (positive) sign for the ‘performance requirements’ attribute when the opt-out was included in the analysis (Table 10). However, when the opt-out was excluded, the results gave the expected positive sign. The coefficients in both analyses were not statistically significant. The positive sign of the attribute when the opt-out was included in the analysis suggested that respondents preferred capitation payments which had performance requirements. This contradicted the qualitative study results that suggested that performance requirements were not preferred for capitation payments. It was also the least important capitation attribute according to respondents (Table 11). Additionally, further analysis in which the opt-out was excluded (Additional file 3), gave a negative sign for the performance requirements attribute. Therefore, for these reasons, the attribute was dropped.

Finally, the acceptable/unacceptable question was reworded to make it clear and understandable to the respondents that they were first required to rank all three alternatives and then answer if alternative A and/or alternative B were unacceptable (Table 13). The simplified acceptable/unacceptable question was set to only appear under alternative A and alternative B and not the opt-out.

**Table 13:**
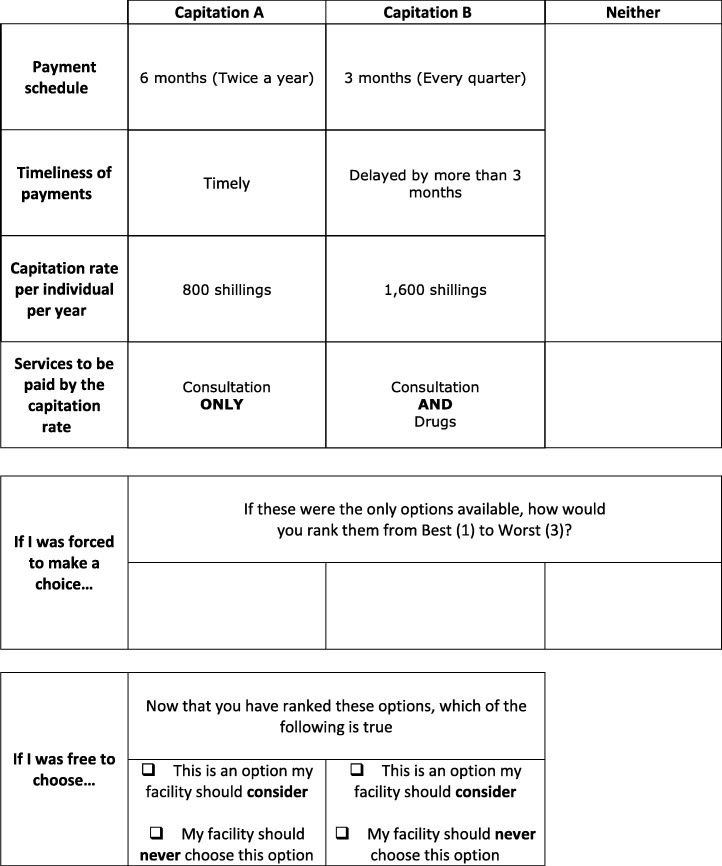
Sample final DCE survey choice task

## Discussion

Health-related DCEs rarely comprehensively conduct and report the attribute and level selection process [10]. This is because of the lack of systematic guidelines on how to do so [16]. However, few researchers such as Helter and Boehler [21] have proposed frameworks to guide the attribute development process. We followed Helter and Boehler’s four-stage framework to rigorously conduct and report the process of attribute development and level selection for a DCE to elicit the preferences of health care providers for the attributes of capitation. The process included raw data collection, data reduction, removing inappropriate attributes, and wording of attributes. The whole process resulted in four capitation attributes to be included in the main DCE, namely, payment schedule, timeliness of payments, capitation rate per individual per year, and services to be paid by the capitation rate.

The first two stages, which included a literature review and qualitative study, resulted in a long list of attributes and levels. While other studies used either qualitative studies [15, 61] or literature reviews only, we used a combination of both methods. Using literature reviews alone may lead to omission of some relevant attributes which may, in turn, increase the error variances and introduce bias into the study [7, 11]. Therefore, qualitative studies are advocated for as they help in identifying context-specific attributes that are important to the study respondents [11, 14, 15]. Furthermore, qualitative studies can also help in revealing new attributes not captured in literature. In our study, the literature review identified conceptual attributes while the qualitative study unearthed context-specific attributes. Several studies have adopted such strategies [14, 62].

This study engaged experts to reduce the number of attributes and levels. Engaging experts who are not part of the research team is beneficial as it avoids narrowing the focus in the preliminary stages of the study [12]. The approach is also useful when it complements other techniques such as literature reviews and qualitative studies [21].

Additionally, unlike other studies [12, 14], we presented detailed pilot study results including regression coefficients and willingness to accept estimates. We could judge the validity of the DCE by comparing the pilot study estimates with the qualitative study results. The signs of the coefficients of four attributes were expected. We found preferences for capitation schemes that had frequent disbursements, timely payments, higher rates per individual, and paid for basic service packages. Furthermore, respondents made trade-offs. Moreover, the analysis revealed that the payment rate per individual per year and payment schedule were two of the most important capitation attributes. This is because higher rates meant more revenue to health care providers and regular payment schedules ensured that facilities could plan and budget [30, 56]. Though there are few DCEs that focussed on health care providers’ preferences for PPMs, Robyn et al. [31] did find similar results in a DCE conducted among health workers in Burkina. Furthermore, Robyn et al. included payment schedule and capitation rate per individual attributes in their actual DCE. However, the study included a ‘performance-based payment’ characteristic which we had dropped from the final list of attributes to be included in the DCE. This was because the analysis of our pilot study results gave an unexpected positive coefficient for the attribute when the opt-out was included and estimates revealed that it was the least important attribute. Studies have demonstrated that capitation incentivises health care providers to compromise performance for example underserving patients [63]. Though Robyn et al. included the attribute in their study as it was important, it was not important in Kenya. Burkina Faso is a different context from Kenya. The current capitation arrangement in Kenya would make health care providers resent performance requirements being attached to the payment mechanism. Piloting of the attributes coupled with a comparison of the results with the qualitative study was vital as we could have misspecified attributes and levels and therefore misinform policy [62].

### Strengths and limitations

This paper has several strengths. First, the study serves as an example of how to rigorously and systematically conduct and report the process of deriving attributes and levels. This improves transparency and makes it reproducible. Secondly, our pilot study results were proof that study participants could consider all information in reaching a decision, place relative importance on the attributes, and make trade-offs. Similar findings were observed by Gomes et al. [64] in their DCE pilot study. Also, the think-aloud exercise employed during the pilot test assisted in gauging respondents understandability of the choice tasks [12].

On the contrary, the study had some limitations. First, the sample size for the pilot study might have been insufficient. This might explain why the coefficients of two attributes were not statically significantly different from zero. Second, we estimated an MNL model which does not relax the IIA assumption. However, we additionally ran a panel MMNL model (Additional file 4) to relax IIA and found that the results were not very different from those from the MNL. Therefore, we used the MNL results to make our decisions as it is a stable model with a small sample size. Third, the qualitative study focussed on the views of NHIF-accredited health care providers leaving out those who were not NHIF-accredited. Nonetheless, the pilot study included both accredited and non-accredited providers.

## Conclusion

The paper contributes to DCE literature by rigorously conducting and reporting the process of attribute development and level selection. Researchers should embrace the practice as it improves transparency and helps in judging the “quality” of the DCE.

## Supplementary information

**Additional file 1.** [Questionnaire]. Interview guide. Qualitative study interview guide. (PDF 222 kb)

**Additional file 2.** [Pilot study questionnaire]. Questionnaire. Pilot study choice experiment questionnaire. (PDF 850 kb)

**Additional file 3.** [Forced choice statistics]. Main effects MNL model estimates (forced choice – opt-out not included). Table showing the MNL model forced choice estimates. (DOCX 15 kb)

**Additional file 4.** [Panel MMNL preference estimates]. Panel MMNL model main effects preference estimates. Table showing the panel MMNL model main effects preference estimates. (DOCX 14 kb)

## Data Availability

The data generated and analysed during the qualitative study are not publicly available due to them containing information that could compromise research participant privacy. However, the transcripts are available from the corresponding author [MO] or [EB] on reasonable request. The quantitative dataset analysed for this study are stored in the KEMRI-Wellcome Trust Research Programme (KWTRP) Data Repository 10.7910/DVN/AGIPLL and can be made available via written request to the corresponding author [MO] or [EB].

## Abbreviations

CMC: The Choice Modelling Centre at the University of Leeds
DCE: Discrete Choice Experiment
HMT: Health Management Team
KEMRI: Kenya Medical Research Institute
MNL: Multinomial Logit Model
NGO: Non-Governmental Organisation
NHIF: National Hospital Insurance Fund
PPM: Provider Payment Mechanism
RESYST: Resilient and Responsive Health Systems
RUT: Random Utility Theory
SERU: Scientific and Ethics Review Unit
UHC: Universal Health Coverage
WTA: Willingness to Accept
