# Supplementary material for: Attribute development and level selection for a discrete choice experiment to elicit the preferences of health care providers for capitation payment mechanism in Kenya
Source: Health Econ Rev. 2019 Oct 30;9:30. doi: 10.1186/s13561-019-0247-5 (PMC6822414; doi:10.1186/s13561-019-0247-5)
Supplement: Supplementary file 1 — Additional file 1. [Questionnaire]. Interview guide. Qualitative study interview guide. (PDF 222 kb) [file 13561_2019_247_MOESM1_ESM.pdf]

SEMI STRUCTURED INTERVIEW GUIDE WITH HEALTH CARE PROVIDER MANAGEMENT TEAM MEMBERS.

1.0 Part A

1.1 Introductory questions

**Interview number**	
**Date of interview**	
**Name of county**	
**Name of facility**	
**Type of facility (Public, Private, Faith-based/Mission)**	
**Role of interviewee (Medical superintendent, matron, pharmacist, accountant, administrator)**	
**Gender of interviewee**	

[Interviewer to describe the concepts of provider payment mechanisms/methods]

Provider payment mechanisms are methods by which the facility is paid for the services it delivers (e.g. by NHIF, private insurers, county government, national government etc)

2.0 Part B

2.1 Awareness and understanding of Provider Payment Mechanisms (PPMs)

2.1.1 Which types of PPMs do you know of?

Probe: (Line-item, global budget, capitation, fee-for-service, payment-for-performance, etc.)

2.1.2 Which of these provider payment mechanisms are used in this facility? Could you also describe how these payment mechanisms work?

2.1.3 What are the positive aspects of these PPMs used in this facility?Why?

2.1.4 What are the negative aspects of these PPMs used in this facility? Why?

2.1.5 (*Ask this question only if they identify negative aspects in 2.1.4*) What do you think can be done to overcome these challenges? By whom?

3.0 Part C

3.1 **<u>Deriving attributes of PPMS</u>**

3.1.1 If you are to design a PPM for health care providers, what aspects or characteristics of this ideal payment method would you consider very important? **(***Ask why for each attribute identified*)?

Probe: (*only if respondent is finding difficulties in answering the questions*)

- ✓ Range of services to covered
- ✓ Adequacy or sufficiency of the PPM
- ✓ Flexibility in use of the funds
- ✓ Predictability of the PPM
- ✓ Performance requirements of the PPM
- ✓ Payment schedule of the PPM
- ✓ Complexity of accountability mechanism of the PPM

3.1.2 Other than these elements/features (*Mention the features identified by the respondent*), are there any other characteristics of PPMs you would consider important? What are they? And why?

3.1.3 Among the characteristics you have mentioned above (***Interviewer:*** *state all the characteristics as identified by the respondent*) which do you consider most important? (*Probe to rank the characteristics identified)* Why?

3.1.4 As a health care provider, would designing PPMs to your preferences influence the way you deliver services? (*Probe for availability, quality, and efficiency in service delivery*) Why?

4 Part D

4.0 **<u>Deriving attribute-levels</u>**

I will now ask you more specific questions about the important elements/features (Mention the features) of PPMs and how each feature should vary. Specifically, we will use Capitation and Fee-for-service as examples to understand the alternatives in the range of services to be covered, the payment schedule, the autonomy in use of the funds, predictability in payment, adequacy of the rate/total funds, complexity of accountability mechanisms, and whether you would prefer performance requirements attached to the PPM.

4.1 **<u>Range of services to be covered</u>**

4.1.1 Which services would you want to be covered under capitation? Why?

4.1.2 Which services would you want to be covered under fee-for-service? Why?

Some PPMs pay for grouped or bundled services while others reimburse for each individual service offered. For example, capitation payment maybe designed to pay for a range of grouped/bundled services (consultation, drugs, lab tests, other diagnostics) while fee-for-service reimburses for each individual service. If you were to design a payment mechanism for an insurance scheme:

4.1.3 Would you prefer to bundle services (i.e. all services are paid for as a group) or would you unbundle some services (i.e. charge the insurer for each individual service)? Why?

4.1.4 If you are forced to bundle/group together two or more services, which ones would you group? Why?

Probe for – Consultation, drugs, lab tests and other diagnostics

4.2 Payment schedule

4.2.1 How often would you like to be paid for the services you provide under capitation? Why?

4.2.2 Apart from (*mention the period the interviewee gave)*, what other alternatives would you prefer and why?

4.2.3 In fee-for-service, what would be your preferred waiting time from the day you submit the claims to when you get the payment? Why?

4.2.4 Apart from (*mention the period the interviewee gave*), what other alternatives would you prefer and why?

4.3 Predictability of amount and time of payment

4.3.1 Now, considering the ideal PPM, what is your opinion about the predictability of the amount of money to be received? (*Knowing the exact amount of money to be received*) Why?

4.3.2 What is your opinion on the predictability of the time of payment? Why?

4.4 Adequacy of the PPM rate to cover the cost of services

4.4.1 To what extent would you like the rates under capitation & fee for service to cover the costs of services sought by patients? Why?

Probe: full or percentage of costs

4.4.2 What is your opinion about co-payments by the patient? Why?

The capitation payment is based on numbers and risk pooling – Where it is assumed that not all people will fall sick at the same time. Therefore, the high costs incurred by the sick are accommodated by those who are well.

4.4.3 What do you think will be a reasonable amount that you should receive for a family per year under capitation? Why?

4.4.4 How would you like a PPM rate be determined/set?

Probe: Before service delivery – prospectively vs After service delivery - retrospectively) Why?
Prices reflecting market value
Payments based on inputs used or output produced

4.5 Flexibility/autonomy in use of funds from capitation and fee-for-service

4.5.1 What sort of freedom would you want to have in using the funds from *capitation and fee-for-service* **?** Why?

Probes:
- ✓ *Free to use funds for anything or restricted for specific use*

4.6 Accountability mechanisms associated with the PPM

4.6.1 What reporting requirements would you prefer to be associated with capitation & fee-for-service? Why?

4.6.2 How would you prefer the level of monitoring/reporting requirements under capitation & fee-for-service to be? Why?
- ✓ *How strict would you prefer the reporting requirements to be?*
- ✓ *What level of complexity would you prefer in the reporting requirements of PPMs?*

4.7 Performance requirements of the PPM

4.7.1 In your opinion would you prefer a PPM whose rate is based on performance (i.e. performance based payment) or one that has a constant rate regardless of performance? Why?

4.7.2 What performance requirements would you prefer to be associated with capitation and fee-for-service? (Quantity or Quality) Why?

5 **Part E**

5.0 **Summary**

5.3 Is there any other characteristic of PPMs that you like to add? If so, which element? Why?

Thank you very much
