# Supplementary material for: Attribute development and level selection for a discrete choice experiment to elicit the preferences of health care providers for capitation payment mechanism in Kenya
Source: Health Econ Rev. 2019 Oct 30;9:30. doi: 10.1186/s13561-019-0247-5 (PMC6822414; doi:10.1186/s13561-019-0247-5)
Supplement: Supplementary file 2 — Additional file 2. [Pilot study questionnaire]. Questionnaire. Pilot study choice experiment questionnaire. (PDF 850 kb) [file 13561_2019_247_MOESM2_ESM.pdf]

A CHOICE EXPERIMENT TO ELICIT THE PREFERENCES OF HEALTH CARE PROVIDERS FOR THE ATTRIBUTES OF CAPITATION PAYMENT MECHANISM.

Section A. Information about the health facility *(To be completed by researcher)*

Questionnaire number: ________________________ Date and time: ________________________
County: ______________________________________ Sub county: ___________________________
Health facility name: ___________________________ Health facility id: ______________________

1. Health facility ownership *(Tick one of the boxes below)*

   ☐Private-for-profit ☐ Public ☐Faith-based organisation ☐NGO

2. Health facility KEPH (Kenya Essential Package for Health) level

   ☐Level 3 ☐Level 4 ☐Level 5 ☐Level 6

3. NHIF accreditation status? *(Tick one of the boxes below)*

   ☐Currently accredited ☐Used to be accredited ☐Never accredited

4. If the answer is currently or used to be accredited in question 3, list the NHIF schemes *(You can tick multiple options*). Otherwise, skip and move to question 5.

   ☐Civil Servants' scheme ☐National Scheme ☐Linda Mama ☐Managed schemes (County government and parastal schemes) ☐Health Insurance Subsidy Program for the orphaned and vulnerable children (OVC) ☐Health Insurance Subsidy Program for the Elderly and those living with disability

5. Private health insurance accreditation *(Tick one of the boxes below)*

   ☐Currently accredited ☐Used to be accredited ☐Never accredited

   (If the answer is currently or used to be accredited, list the private health insurers)

   ____

   ____

6. Does the facility currently or used to receive capitation payments from NHIF or any other health insurer?

   ☐Currently receives ☐Used to receive ☐Never received

   (If the answer is currently or used to receive, list the insurers)

   ____

   ____

Section B. Information about you *(To be completed by the respondent)*

1. What is your gender? (*Tick one of the boxes below.)*

   ☐Male ☐Female

2. How old were you at your last birth day? (*Provide age in years*) _____________

3. What is your profession? (e.g. Medical doctor, nurse, accountant etc.) _____________________

4. What is your job title/designation? (e.g. Hospital Administrative Officer, Medical Superintendent, Pharmacist, Nurse-in-charge etc.) ______________________________________

5. How many years of work experience do you have? ______________________________

Section C. Introduction to capitation *(For the respondent to read)*

Capitation is a payment method where health facilities are paid a fixed amount of money by health insurers (e.g. NHIF) for every member registered to that facility to provide services over a fixed period. The funds are paid in advance (i.e. before health care services are offered).

Capitation is based on numbers and risk pooling. It is based on numbers because the total amount of money a health facility is paid depends on the number of health insurance beneficiaries registered to that facility. If a facility has 1000 registered health insurance beneficiaries, and the insurer pays a rate of KES 1200 per member per year, then the facility expects to receive (1200 X 1000) KES 1,200,000 per year. Therefore, a facility with more registered health insurance beneficiaries would receive a higher amount as compared to one that has fewer members. For example, a facility with 1500 members will get (1200 X 1500) KES 1,800,000 while a facility with 500 members will receive KES 600,000 per year.

In addition, capitation has an aspect of risk pooling in that not all the registered health insurance beneficiaries will seek care at the same time. As a result, the costs of care of those who visit the facility are covered from the total capitation amount paid by the health insurer (e.g. KES 1,200,000 for the 1000 registered members) and not the rate per individual (e.g. 1,200). For example, if 300 out of the 1,000 registered members seek care at the facility over a one-year period, and each spends a total of KES 2,500, then the total costs incurred by the facility are (KES 2,500 X 300) KES 750,000. This means that the facility still makes a profit of (1,200,000 – 750,000) KES 450,000.

Section C. Choice experiment *(For the respondent to read)*

Imagine you are to sign a contract with a health insurer (e.g. NHIF) to provide services to its members. You are to be paid using the capitation model. You will be presented with eight sets of tasks, each representing two alternatives of a possible capitation model of payment and a no choice alternative. These alternatives differ only by the levels of the following characteristics:

1. **Payment Schedule** – frequency of capitation disbursements which takes the form of either 1 month (Monthly), 3 months (Quarterly), 6 months (Bi annually - Twice a year), or 12 Months (Annually – Once a year)
2. **Timeliness of payments** – Whether the capitation disbursements are made on time (by the agreed date) or not.
3. **Capitation rate per individual per year**. This is the amount of money paid by a health insurer for an individual to cover the costs of services provided by the health facility to that person. This takes the form of KES 1200, 2400, 3600, and 4800 per individual per year.
4. **Services to be paid by the capitation rate**. This refers to the number and type of services that the insurer pays for by capitation. For example, the insurer might pay for consultation, drugs, and lab tests by capitation, OR pay for consultation and drugs only by capitation, and pay for lab tests separately using another method such as fee for service.
5. **Performance requirements.** Performance requirements might come attached to capitation payments. For example, a health facility might receive a base capitation rate only, or get the base capitation rate plus an additional bonus when they improve the quality of services that they offer to patients.

For each task, you will be asked to rank your preferred choice with (1) representing your best choice, (2) second best, and (3) your worst. Then, for the alternatives that you ranked (2) and (3), you will be prompted to indicated which one you find unacceptable (i.e. you would never choose that alternative?). Tick the remaining alternative as acceptable.

Practice choice tasks

Practice scenario 1 *(Researcher to use this as a demonstration)*

	**Capitation A**	**Capitation B**	**None**
**Payment schedule**	6 months	3 months	
**Timeliness of payments**	Timely	Delayed	
**Capitation rate per individual per year**	3,600 shillings	4,800 shillings	
**Services to be paid by the capitation rate**	Capitation rate pays for consultation only. (Hospital claims and is paid for lab tests and drugs separately by the insurer/NHIF).	Capitation rate pays for consultation and drugs only (Hospital claims and is paid for lab tests separately by the insurer/NHIF).	
**Performance requirements**	Hospital receives base/fixed capitation rate	Hospital receives base/fixed capitation rate + bonus for improved performance (e.g improved quality).	
**Please rank your preferred choice Best(1) to Worst(3)**	☐	☐	☐
**Among the alternatives that you ranked (2) and (3) which one is unacceptable to you (i.e. you would never choose that alternative?)**	☐ Acceptable<br>☐ Unacceptable	☐ Acceptable<br>☐ Unacceptable	☐ Acceptable<br>☐ Unacceptable

Practice Scenario 2 *(To be completed by respondent)*

	**Capitation A**	**Capitation B**	**None**
**Payment schedule**	1 month	12 months	
**Timeliness of payments**	Timely	Delayed	
**Capitation rate per individual per year**	1,200 shillings	4,800 shillings	
**Services to be paid by the capitation rate**	Capitation rate pays for consultation only. (Hospital claims and is paid for lab tests and drugs separately by the insurer/NHIF).	Capitation rate pays for consultation, lab tests, and drugs	
**Performance requirements**	Hospital receives base/fixed capitation rate + bonus for improved performance (e.g. improved quality).	Hospital receives base/fixed capitation rate	
**Please rank your preferred choice Best(1) to Worst(3)**	☐	☐	☐
**Among the alternatives that you ranked (2) and (3) which one is unacceptable to you (i.e. you would never choose that alternative?)**	☐ Acceptable<br>☐ Unacceptable	☐ Acceptable<br>☐ Unacceptable	☐ Acceptable<br>☐ Unacceptable

Section D: Choice tasks *(To be completed by respondent)*

Scenario 1

	**Capitation A**	**Capitation B**	**None**
**Payment schedule**	1 month	12 months	
**Timeliness of payments**	Delayed	Timely	
**Capitation rate per individual per year**	2,400 shillings	3,600 shillings	
**Services to be paid by the capitation rate**	Capitation rate pays for consultation and lab tests only. (Hospital claims and is paid for drugs separately by the insurer/NHIF).	Capitation rate pays for consultation and drugs only (Hospital claims and is paid for lab tests separately by the insurer/NHIF).	
**Performance requirements**	Hospital receives base/fixed capitation rate	Hospital receives base/fixed capitation rate + bonus for improved performance (e.g improved quality).	
**Please rank your preferred choice Best(1) to Worst(3)**	☐	☐	☐
**Among the alternatives that you ranked (2) and (3) which one is unacceptable to you (i.e. you would never choose that alternative?)**	☐ Acceptable<br>☐ Unacceptable	☐ Acceptable<br>☐ Unacceptable	☐ Acceptable<br>☐ Unacceptable

Scenario 2

	**Capitation A**	**Capitation B**	**None**
**Payment schedule**	12 months	1 month	
**Timeliness of payments**	Delayed	Timely	
**Capitation rate per individual per year**	3,600 shillings	2,400 shillings	
**Services to be paid by the capitation rate**	Capitation rate pays for consultation and drugs only (Hospital claims and is paid for lab tests separately by the insurer/NHIF).	Capitation rate pays for consultation and lab tests only. (Hospital claims and is paid for drugs separately by the insurer/NHIF).	
**Performance requirements**	Hospital receives base/fixed capitation rate	Hospital receives base/fixed capitation rate + bonus for improved performance (e.g. improved quality).	
**Please rank your preferred choice Best(1) to Worst(3)**	☐	☐	☐
**Among the alternatives that you ranked (2) and (3) which one is unacceptable to you (i.e. you would never choose that alternative?)**	☐ Acceptable<br>☐ Unacceptable	☐ Acceptable<br>☐ Unacceptable	☐ Acceptable<br>☐ Unacceptable

Scenario 3

	Capitation A	Capitation B	None
**Payment schedule**	1 month	12 months	
**Timeliness of payments**	Timely	Delayed	
**Capitation rate per individual per year**	3,600 shillings	2,400 shillings	
**Services to be paid by the capitation rate**	Capitation rate pays for consultation and lab tests only. (Hospital claims and is paid for drugs separately by the insurer/NHIF).	Capitation rate pays for consultation and drugs only (Hospital claims and is paid for lab tests separately by the insurer/NHIF).	
**Performance requirements**	Hospital receives base/fixed capitation rate	Hospital receives base/fixed capitation rate + bonus for improved performance (e.g. improved quality).	
**Please rank your preferred choice Best(1) to Worst(3)**	☐	☐	☐
**Among the alternatives that you ranked (2) and (3) which one is unacceptable to you (i.e. you would never choose that alternative?)**	☐ Acceptable<br>☐ Unacceptable	☐ Acceptable<br>☐ Unacceptable	☐ Acceptable<br>☐ Acceptable

Scenario 4

	Capitation A	Capitation B	None
**Payment schedule**	6 months	3 months	
**Timeliness of payments**	Delayed	Timely	
**Capitation rate per individual per year**	4,800 shillings	1,200 shillings	
**Services to be paid by the capitation rate**	Capitation rate pays for consultation, lab tests, and drugs	Capitation rate pays for consultation only. (Hospital claims and is paid for lab tests and drugs separately by the insurer/NHIF).	
**Performance requirements**	Hospital receives base/fixed capitation rate + bonus for improved performance (e.g. improved quality).	Hospital receives base/fixed capitation rate	
**Please rank your preferred choice Best(1) to Worst(3)**	☐	☐	☐
**Among the alternatives that you ranked (2) and (3) which one is unacceptable to you (i.e. you would never choose that alternative?)**	☐ Acceptable<br>☐ Unacceptable	☐ Acceptable<br>☐ Unacceptable	☐ Acceptable<br>☐ Unacceptable

Scenario 5

	**Capitation A**	**Capitation B**	**None**
**Payment schedule**	12 months	1 month	
**Timeliness of payments**	Timely	Delayed	
**Capitation rate per individual per year**	2,400 shillings	3,600 shillings	
**Services to be paid by the capitation rate**	Capitation rate pays for consultation and drugs only (Hospital claims and is paid for lab tests separately by the insurer/NHIF).	Capitation rate pays for consultation and lab tests only. (Hospital claims and is paid for drugs separately by the insurer/NHIF).	
**Performance requirements**	Hospital receives base/fixed capitation rate	Hospital receives base/fixed capitation rate + bonus for improved performance (e.g. improved quality).	
**Please rank your preferred choice Best(1) to Worst(3)**	☐	☐	☐
**Among the alternatives that you ranked (2) and (3) which one is unacceptable to you (i.e. you would never choose that alternative?)**	☐ Acceptable<br>☐ Unacceptable	☐ Acceptable<br>☐ Unacceptable	☐ Acceptable<br>☐ Unacceptable

Scenario 6

	Capitation A	Capitation B	None
**Payment schedule**	3 months	6 months	
**Timeliness of payments**	Timely	Delayed	
**Capitation rate per individual per year**	4,800 shillings	1,200 shillings	
**Services to be paid by the capitation rate**	Capitation rate pays for consultation only. (Hospital claims and is paid for lab tests and drugs separately by the insurer/NHIF).	Capitation rate pays for consultation, lab tests, and drugs	
**Performance requirements**	Hospital receives base/fixed capitation rate + bonus for improved performance (e.g. improved quality).	Hospital receives base/fixed capitation rate	
**Please rank your preferred choice Best(1) to Worst(3)**	☐	☐	☐
**Among the alternatives that you ranked (2) and (3) which one is unacceptable to you (i.e. you would never choose that alternative?)**	☐ Acceptable<br>☐ Unacceptable	☐ Acceptable<br>☐ Unacceptable	☐ Acceptable<br>☐ Unacceptable

Scenario 7

	Capitation A	Capitation B	None
**Payment schedule**	6 months	3 months	
**Timeliness of payments**	Timely	Delayed	
**Capitation rate per individual per year**	1,200 shillings	4,800 shillings	
**Services to be paid by the capitation rate**	Capitation rate pays for consultation, lab tests, and drugs	Capitation rate pays for consultation only. (Hospital claims and is paid for lab tests and drugs separately by the insurer/NHIF).	
**Performance requirements**	Hospital receives base/fixed capitation rate + bonus for improved performance (e.g. improved quality).	Hospital receives base/fixed capitation rate	
**Please rank your preferred choice Best(1) to Worst(3)**	☐	☐	☐
**Among the alternatives that you ranked (2) and (3) which one is unacceptable to you (i.e. you would never choose that alternative?)**	☐ Acceptable<br>☐ Unacceptable	☐ Acceptable<br>☐ Unacceptable	☐ Acceptable<br>☐ Unacceptable

Scenario 8

	Capitation A	Capitation B	None
**Payment schedule**	3 months	6 months	
**Timeliness of payments**	Delayed	Timely	
**Capitation rate per individual per year**	1,200 shillings	4,800 shillings	
**Services to be paid by the capitation rate**	Capitation rate pays for consultation only. (Hospital claims and is paid for lab tests and drugs separately by the insurer/NHIF).	Capitation rate pays for consultation, lab tests, and drugs	
**Performance requirements**	Hospital receives base/fixed capitation rate + bonus for improved performance (e.g. improved quality).	Hospital receives base/fixed capitation rate	
**Please rank your preferred choice Best(1) to Worst(3)**	☐	☐	☐
**Among the alternatives that you ranked (2) and (3) which one is unacceptable to you (i.e. you would never choose that alternative?)**	☐ Acceptable<br>☐ Unacceptable	☐ Acceptable<br>☐ Unacceptable	☐ Acceptable<br>☐ Unacceptable
