# Supplementary material for: Attribute development and level selection for a discrete choice experiment to elicit the preferences of health care providers for capitation payment mechanism in Kenya
Source: Health Econ Rev. 2019 Oct 30;9:30. doi: 10.1186/s13561-019-0247-5 (PMC6822414; doi:10.1186/s13561-019-0247-5)
Supplement: Supplementary file 3 — Additional file 3. [Forced choice statistics]. Main effects MNL model estimates (forced choice – opt-out not included). Table showing the MNL model forced choice estimates. (DOCX 15 kb) [file 13561_2019_247_MOESM3_ESM.docx]

Additional file 3: Main effects MNL model estimates (forced choice – opt-out excluded from the analysis)

|  |  | Preference estimates | Willingness to accept (WTA) |
| --- | --- | --- | --- |
| **Attributes** | **Levels** | **Coefficient**  **(robust se)** | **value**  **(robust se)** |
| Payment schedule | 1 month | -0.0971***  (0.0252) | 342.5017***  (86.3976) |
|  | 3 months |  |  |
|  | 6 months |  |  |
|  | 12 months |  |  |
| Timeliness of payments | Delayed | 0.4990***  (0.1469) | -1759.9235**  (583.3697) |
|  | Timely |  |  |
| Capitation rate per individual per year | 1200 shillings | 0.0003**  (0.0001) |  |
|  | 2400 shillings |  |  |
|  | 3600 shillings |  |  |
|  | 4800 shillings |  |  |
| Services to be paid by the capitation rate | Capitation rate pays for consultation only. | -0.0583  (0.0852) | 205.6377  (299.9566) |
|  | Capitation rate pays for consultation and drugs only |  |  |
|  | Capitation rate pays for consultation and lab tests only |  |  |
|  | Capitation rate pays for consultation, lab tests, and drugs |  |  |
| Performance requirements | Hospital receives base/fixed capitation rate | -0.0543  (0.1223) | 191.5855  (430.4992) |
|  | Hospital receives base/fixed capitation rate + bonus for improved performance (e.g. improved quality) |  |  |
| Constant (Alternative A) |  | -0.1931  (0.1180) | 681.0577  (435.7841) |
| **Model fit statistics** |  |  |  |
| Log likelihood at convergence | -170.5351 |  |  |
| Log likelihood (final) | -135.975 |  |  |
| Adjusted rho-squared at convergence | 0.17 |  |  |
| Akaike Information Criterion | 283.95 |  |  |
| Bayesian Information Criterion | 305.03 |  |  |
| observations | 248 |  |  |
| number of decision makers (n) | 31 |  |  |
| s.e. - Robust standard errors in parenthesis. Asterisks denote statistical significance at *** 0.1%, ** 1%, and *5% level. | | | |
