# Supplementary material for: Attribute development and level selection for a discrete choice experiment to elicit the preferences of health care providers for capitation payment mechanism in Kenya
Source: Health Econ Rev. 2019 Oct 30;9:30. doi: 10.1186/s13561-019-0247-5 (PMC6822414; doi:10.1186/s13561-019-0247-5)
Supplement: Supplementary file 4 — Additional file 4. [Panel MMNL preference estimates]. Panel MMNL model main effects preference estimates. Table showing the panel MMNL model main effects preference estimates. (DOCX 14 kb) [file 13561_2019_247_MOESM4_ESM.docx]

Additional file 4: Panel MMNL model main effects preference estimates

|  | **Opt-out included** | | **Opt-out excluded (forced choice)** | |
| --- | --- | --- | --- | --- |
| **Attributes** | **Coefficient** | **Robust se** | **Coefficient** | **Robust se** |
| Payment schedule μ | -0.1358** | 0.0476 | -0.1410*** | 0.0418 |
| Payment schedule σ | 0.1573** | 0.0484 | 0.1469*** | 0.0443 |
| Timeliness of payments μ | 0.6655** | 0.2118 | 0.7051*** | 0.2005 |
| Timeliness of payments σ | 0.4408 | 0.5227 | 0.5784 | 0.4435 |
| Capitation rate per individual per year μ | 0.0004*** | 0.0001 | 0.0004*** | 0.0001 |
| Services to be paid by the capitation rate μ | -0.0621 | 0.1206 | -0.0715 | 0.1163 |
| Services to be paid by the capitation rate σ | 0.4650* | 0.233 | 0.4363** | 0.1670 |
| Performance requirements μ | 0.0690 | 0.1491 | -0.079 | 0.1628 |
| Performance requirements σ | -0.0102 | 0.037 | 0.0845 | 0.2752 |
| Opt-out μ | -1.6165 | 0.9333 | - | - |
| Opt-out σ | 2.8979*** | 0.5508 | - | - |
| Constant μ (Alternative A) | - | - | -0.2992 | 0.1897 |
| Constant σ (Alternative A) | - | - | 0.0048 | 0.0276 |
| **Model fit statistics** |  | |  | |
| Log likelihood at convergence | -250.6159 | | -170.5351 | |
| Log likelihood (final) | -189.009 | | -129.1583 | |
| Adjusted rho-squared at convergence | 0.2 | | 0.18 | |
| Akaike Information Criterion | 400.02 | | 280.32 | |
| Bayesian Information Criterion | 438.67 | | 318.96 | |
| Observations | 248 | | 248 | |
| Draws (Halton) | 1000 | | 1000 | |
| Number of decision makers (n) | 31 | | 31 | |
| Se denotes robust standard errors. Asterisks denote *** p<0.001, ** p<0.01, * p<0.05. μ denotes the mean while σ is the standard deviation. All attributes were random following a normal distribution except the ‘capitation rate per individual per year’ attribute which was non-random | | | | |
